# *Pseudomonas aeruginosa* rugose small-colony variants evade host clearance, are hyper-inflammatory, and persist in multiple host environments

**DOI:** 10.1371/journal.ppat.1006842

**Published:** 2018-02-02

**Authors:** Matthew J. Pestrak, Sarah B. Chaney, Heather C. Eggleston, Sheri Dellos-Nolan, Sriteja Dixit, Shomita S. Mathew-Steiner, Sashwati Roy, Matthew R. Parsek, Chandan K. Sen, Daniel J. Wozniak

**Affiliations:** 1 Department of Microbial Infection and Immunity, The Ohio State University, Columbus, Ohio, United States of America; 2 Department of Surgery, The Ohio State University, Columbus, Ohio, United States of America; 3 Department of Microbiology, University of Washington, Seattle, Washington, United States of America; University of North Carolina at Chapel Hil, UNITED STATES

## Abstract

*Pseudomonas aeruginosa* causes devastating infections in immunocompromised individuals. Once established, *P*. *aeruginosa* infections become incredibly difficult to treat due to the development of antibiotic tolerant, aggregated communities known as biofilms. A hyper-biofilm forming clinical variant of *P*. *aeruginosa*, known as a rugose small-colony variant (RSCV), is frequently isolated from chronic infections and is correlated with poor clinical outcome. The development of these mutants during infection suggests a selective advantage for this phenotype, but it remains unclear how this phenotype promotes persistence. While prior studies suggest RSCVs could survive by evading the host immune response, our study reveals infection with the RSCV, PAO1Δ*wspF*, stimulated an extensive inflammatory response that caused significant damage to the surrounding host tissue. In both a chronic wound model and acute pulmonary model of infection, we observed increased bacterial burden, host tissue damage, and a robust neutrophil response during RSCV infection. Given the essential role of neutrophils in *P*. *aeruginosa*-mediated disease, we investigated the impact of the RSCV phenotype on neutrophil function. The RSCV phenotype promoted phagocytic evasion and stimulated neutrophil reactive oxygen species (ROS) production. We also demonstrate that bacterial aggregation and TLR-mediated pro-inflammatory cytokine production contribute to the immune response to RSCVs. Additionally, RSCVs exhibited enhanced tolerance to neutrophil-produced antimicrobials including H_2_O_2_ and the antimicrobial peptide LL-37. Collectively, these data indicate RSCVs elicit a robust but ineffective neutrophil response that causes significant host tissue damage. This study provides new insight on RSCV persistence, and indicates this variant may have a critical role in the recurring tissue damage often associated with chronic infections.

## Author summary

Bacteria evolve rapidly, which can often lead to the development of unique traits better suited for survival in a harsh environment. This phenomenon can be observed in the human host environment during infection with the bacteria *Pseudomonas aeruginosa*, which is particularly prone to diversification. One variant of *P*. *aeruginosa* that commonly develops is a rugose small-colony variant (RSCV), and the frequency of its development indicates it is well adapted for survival in the host. While it is well established that RSCVs overproduce protective biofilm matrix materials, it remains unclear how they survive in the host and their effect on the immune response. In this study, we demonstrate RSCVs are better adapted to multiple host environments and cause more severe infections compared to their parental counterparts. Furthermore, we determined RSCVs elicit a robust inflammatory response from neutrophils, while avoiding many of their various killing mechanisms. Our study indicates the RSCV phenotype provides *P*. *aeruginosa* with enhanced tolerance to host defenses, and that RSCVs may contribute to host tissue damage typically associated with chronic infection.

## Introduction

*Pseudomonas aeruginosa* is one of the most common causes of nosocomial infections, and it is consistently linked to poor clinical outcome [1,2]. These infections are particularly prevalent in immunocompromised patients with indwelling medical devices or wounds, such as surgical sites, burn wounds, and pressure ulcers [1,3,4]. *P*. *aeruginosa* is also the most common cause of devastating chronic pulmonary infections in patients with the genetic disease cystic fibrosis (CF). This organism is isolated from nearly 80% of CF individuals, and is correlated with respiratory failure and death [5]. Most of the pulmonary pathology associated with CF is due to increased susceptibility to bacterial infection and a prolonged, recurrent inflammatory response in the lung ultimately leading to tissue damage and fatal loss of lung function [6–8]. Similarly, chronically infected skin wounds are arrested in the inflammatory stage of wound healing, and large numbers of immune cells localize to the wound site [9]. *P*. *aeruginosa* effectively survives in the host despite a robust inflammatory response, indicating this bacterium is well adapted for evading host clearance.

*P*. *aeruginosa* utilizes a variety of mechanisms to survive in the host, including the formation of aggregated communities known as biofilms [5,10]. During biofilm formation, *P*. *aeruginosa* encases itself in a matrix of various molecules including exopolysaccharides (ePS), proteins, and extracellular DNA. The biofilm matrix protects the bacteria from antimicrobial treatment and host immune clearance leading to chronic infection [10–12]. Biofilm formation requires significant energy input by the bacterium and is therefore tightly regulated. While it is not yet fully understood how this process is controlled, the secondary messenger molecule cyclic dimeric (3’5’) guanosine monophosphate (c-di-GMP) is a key regulator in the transition from planktonic to biofilm growth [13,14]. This molecule activates the production of two critical biofilm ePS, Psl and Pel [15–17], which have many critical functions for the developing biofilm, including surface attachment, structural roles, and resistance to antibiotics and immune clearance [11,18–20].

*P*. *aeruginosa* is a highly adaptable organism, and the hostile environment created by a chronic inflammatory response promotes adaptation and diversification [21]. One type of variant frequently isolated from the sputum of CF patients is known as a rugose small-colony variant (RSCV) [22,23]. The RSCV phenotype arises through mutations that cause overproduction of c-di-GMP resulting in hyper-biofilm forming strains that form dense bacterial aggregates by producing excessive amounts of Pel and Psl [7,23,24]. The presence of RSCVs has important clinical implications and is associated with prolonged antibiotic treatment and poor clinical outcome [22]. Considering RSCVs are typically isolated at the late-stages of infection, it has been proposed that these variants are selected for due to a state of low virulence and slow growth rate [7]. To date RSCV infection studies have consistently shown that this phenotype promotes persistence, but the underlying mechanisms remains unclear [24–26].

In this study, we compare infection with the *P*. *aeruginosa* prototypical strain PAO1 and an isogenic RSCV strain PAO1Δ*wspF* to elucidate how this phenotype affects the immune response and promotes persistence. Prior studies determined the loss of the methylesterase WspF results in continuous WspA signaling and subsequent activation of the diguanylate cyclase WspR [27]. Therefore, loss of WspF leads to overproduction of c-di-GMP and various biofilm matrix components [23,27]. Herein, we demonstrate PAO1Δ*wspF* caused persistent and severe infections in both a wound and pulmonary infection model, despite a robust host response. *In vitro* neutrophil studies suggest RSCVs resist phagocytosis while stimulating the production of reactive oxygen species (ROS). While biofilm ePS did not directly stimulate neutrophils, bacterial aggregation was sufficient to promote ROS production. Bacterial survival studies indicate RSCVs exhibit enhanced tolerance to various neutrophil-produced antimicrobial factors, including LL-37 and H_2_O_2_. Collectively, this work reveals RSCVs stimulate a robust but ineffective immune response that likely contributes to persistence.

## Methods

### Ethics statement

The Ohio State University Institutional Animal Care and Use Committee (IACUC) under the protocols #2008A0012 (pig) and # 2009A0177 (mouse) preapproved animal procedures carried out in this study. All blood for neutrophil isolation was obtained from healthy human adults. Informed written consent was obtained from all donors and the Ohio State University Institutional Review Board (IRB) (2009H0154) approved all procedures.

### Bacterial growth, culture conditions, and culture normalization

Bacterial strains and plasmids used in this study are listed in Table 1. Cultures of *P*. *aeruginosa* were grown overnight rotating at 37°C in Luria broth without salt (LBNS). Log phase cultures (OD_600_ 0.5–0.8) were used in all experiments by inoculating 100–200μL of overnight culture into sterile LBNS and growing for 2-3h rotating at 37°C. Where appropriate, bacterial plasmids were maintained with the addition of 300μg/mL of carbenicillin in LBNS and expression was induced in P_BAD_-CdrAB strains with 1% arabinose[28].

**Table 1:** Bacterial strains and plasmids used in this study.

**Strain**	**Description**	**Source**
PAO1	*P*. *aeruginosa* wild-type	
PAO1Δ*wspF*	Isogenic RSCV strain	J.J. Harrison; [29]
CF127	Clinical RSCV strain	[30]
CF39s	Clinical RSCV strain	[23]
PAO1/pMRP9	Constitutive GFP production	This study
PAO1Δ*wspF*/pMRP9	Constitutive GFP production	This study
PAO1Δ*pelD* P_BAD_-*psl*	Arabinose inducible Psl producing strain	[31]
PAO1Δ*pslBCD* P_BAD_-*pel*	Arabinose inducible Pel producing strain	[31]
PAO1Δ*pel*Δ*psl*	Pel and Psl deficient strain	J.J. Harrison
PAO1Δ*pel*Δ*psl*/pHERD20T	Vector control	This study
PAO1Δ*pel*Δ*psl*/pCdrAB	Forms CdrA dependent aggregates	This study
**Plasmid**	**Description**	**Reference**
pMRP9	Constitutive GFP production	[32]
pCdrAB	CdrAB operon controlled by an arabinose inducible P_BAD_ promoter	[28]

PAO1Δ*wspF* and the other RSCV strains are highly aggregative and were mechanically disrupted with vigorous vortexing and pipetting prior to reading OD_600_. Similar cell numbers were obtained between aggregative and non-aggregative strains when prepared with this method as confirmed by CFU and total protein quantification by BCA (ThermoFisher) comparison (S1 Fig). Prior to all CFU plating, aggregate disruption was ensured by passing 1mL of culture through a 22G needle three times immediately prior to use. This method of disruption was validated using flow cytometry (FACs Canto, BD) to observe the particle sizes of *P*. *aeruginosa* containing a constitutively expressed GFP plasmid (pMRP9) (S1 Fig).

### Porcine full thickness burn wound infection

Porcine infections were carried out as previously described [33]. A total of 10^8^ CFU of *P*. *aeruginosa* PAO1 or PAO1Δ*wspF* in 250μL of PBS was topically applied to the wounds 3 days post full-thickness burning. For each strain, 2 pigs were infected (4 total), and at least 3 full thickness wound strips and 4 (8 mm) punch biopsies were collected 7, 14 and 35 days post-bacterial inoculation (d.p.i.). The strips, containing normal/non-burned skin (approximately 1 cm) on each side of the wound were fixed in 4% formalin prior to being processed and embedded in paraffin for histological analysis. The punch biopsy samples were flash frozen in Optimal Cutting Temperature (OCT) solution and cryosectioned for bacterial immunohistochemical studies. Bacterial burden at 7 d.p.i. and 35 d.p.i. for each animal was assessed by staining 3 frozen sections, as previously described [33]. Bacteria were stained with an α-*Pseudomonas* antibody (1:1000) and an Alexa Fluor 488 goat α-rabbit secondary antibody (1:200). DAPI (1:10000) was used to stain host porcine tissue. Sections were imaged with confocal laser scanning microscopy (CLSM) using an Olympus FV1000 Filter Confocal System. Area of green fluorescence (*P*. *aeruginosa*) was quantified with FIJI to determine the relative bacterial burden in the wound biopsies [34]. CFUs were also quantified from freshly excised punch biopsy tissue homogenate plated on *Pseudomonas* isolation agar (PIA). Three biopsies from each pig at each time point were quantified for CFUs with a limit of detection of 10 CFU/tissue g. Statistical significance was determined by two-way ANOVA followed by Bonferroni’s posthoc tests.

### Murine acute pulmonary infection

Log phase cultures of *P*. *aeruginosa* were pelleted and washed once in sterile phosphate buffered saline (PBS, 1x). Six-week old female BALB/c mice were anesthetized with isoflurane and inoculated via intranasal instillation with 10^8^ bacteria in 30μl volume with either PAO1 or PAO1Δ*wspF*. Uninfected controls were treated with 30μl of sterile PBS. Each treatment group consisted of at least 5 mice. Where indicated, bacterial aggregates in culture were disrupted immediately prior to infection as described above. At 2, 8, 24, and 48h mice were euthanized and lungs were collected in PBS or infused and placed in 10% neutral buffered formalin (NBF) at a ratio of approximately 1:10 (tissue:NBF by volume). Formalin fixed tissues were processed for histopathology after at least 72h of fixation. Lung tissue was weighed and homogenized in 1mL of PBS and centrifuged at 200 x*g* for 3min to remove cellular debris. IL-1β and IL-6 was measured by ELISA (BD Bioscience) according to manufacturer’s instructions. Statistical significance was determined with two-way ANOVA followed by Bonferroni’s posthoc tests.

### Histopathology

All tissue sectioning and staining was done by the Comparative Pathology Core at the Ohio State University College of Veterinary Medicine. Formalin fixed tissues were paraffin embedded, sectioned (5μm) and stained with hematoxylin and eosin (H&E). The slides were scanned with Aperio Slide Scanner, Scan Scope XT, up to 40x resolution and viewed, analyzed and measured using ImageScope Software (Leica Biosystems, Buffalo Grove, IL). For samples from the porcine model, wound closure was quantified in 4 wounds per condition, by measuring the distance between epithelial tongues (leading edges of the closing wound) of mounted H&E stained wound strips, as previously described [35]. For samples from the murine model lung pathology scoring was performed by a blinded pathologist. The pulmonary changes were scored on the following scale: No Significant Changes, Minimal, Moderate, or Severe. Minimal changes were defined by no loss of pulmonary parenchymal architecture and neutrophils rarely identified in clusters. Moderate inflammation was defined by robust suppurative and neutrophilic inflammation without loss of pulmonary architecture. Severe changes included areas of loss of pulmonary architecture with necrosis or consolidation of the pulmonary airways by suppurative inflammation. Surface areas of each scored area in all lung fields were assessed and measured using the ImageScope software and standardized to the total lung surface area available for evaluation.

### Neutrophil isolation

Human neutrophils (PMNs) were obtained from healthy adult donors as described previously [36]. Heparinized blood was layered on Ficoll Hypaque solution and centrifuged at 400x*g* to obtain a PMN rich pellet. The PMN pellet was then suspended in 0.9% NaCl solution, and red blood cells (RBC) were removed by 1.5% dextran sedimentation at 4°C for 20min. The remaining RBCs were lysed with distilled water, and the neutrophils were washed and suspended in Hank’s buffered salt solution (HBSS). Neutrophils were enumerated and viability assessed using a hemocytometer counterstained with membrane exclusion dye, Trypan Blue.

### Neutrophil internalization

Neutrophils were isolated from the peripheral blood of healthy human donors, and phagocytosis was measured using two established methods [36,37]. 1) CLSM: Neutrophils seeded on poly-L-lysine coated coverslips were infected with log phase cultures of PAO1 or PAO1Δ*wspF* (MOI 1 neutrophil to 50 bacteria). The infection was allowed to occur for 30min at 37°C. Coverslips were washed 2 times in PBS and fixed with 4% paraformaldehyde. Extracellular *P*. *aeruginosa* was stained with a rabbit polyclonal α-*Pseudomonas* antibody (1:2500) [36] and an Alexa Fluor 488 goat α-rabbit secondary antibody (1:1000). The neutrophils were permeabilized with methanol and intracellular *P*. *aeruginosa* was stained with the α-*Pseudomonas* antibody (1:2500) and an Alexa Fluor 647 goat α-rabbit secondary antibody (1:1000). Phagocytosis was assessed with CLSM with an Olympus FV1000 Filter Confocal System by counting the number of neutrophils that co-localized with bacteria stained with Alexa Fluor 647 (red) but not Alexa Fluor 488 (green). Three biological replicates using neutrophils from different donors were measured in duplicate. 2) Flow Cytometry: The pMRP9 plasmid was transformed into PAO1 and PAO1Δ*wspF* allowing for constitutive GFP expression in these strains. Log phase cultures were washed once in PBS, and where indicated, bacteria were opsonized in 20% pooled human serum in PBS for 5min at 37 °C. Neutrophils were infected with bacteria (MOI 1 neutrophil to 50 bacteria) for 30min at 37°C. Neutrophils were washed twice in PBS by centrifugation at 2,500x*g* for 2min. Neutrophils were fixed in 4% paraformaldehyde and bacteria that remained extracellular were stained with the α-*Pseudomonas* antibody (1:2500) and goat α-rabbit Alexa Fluor 647 (1:1000) to allow exclusion of neutrophils coated in bacteria that had not necessarily internalized any cells. A BD FACSCanto II flow cytometer (BD Biosciences) and FlowJo 9.0 analysis software was used to calculate the neutrophil population that was GFP+/Alexa647-. Three biological replicates were measured in triplicate and normalized to PAO1 internalization to reduce donor to donor variation. Statistical significance was determined by Student’s t-test.

### Exopolysaccharide isolation and quantification

Psl or Pel was isolated from overnight cultures of PAO1Δ*pel*P_BAD_-*psl*, PAO1Δ*psl*P_BAD_-*pel*, or PAO1Δ*pel*Δ*psl* grown in LBNS with 1% arabinose, as previously described [38,39]. Briefly, cell pellets were boiled in 0.5M EDTA and cell debris was removed by centrifugation. The ePS preparations were treated with 0.5mg/ml of proteinase K (Qiagen) at 60°C for 60min and then at 80°C for 30min to deactivate the enzyme. Total carbohydrate was quantified in each sample by phenol-sulfuric acid assay [40]. Final Psl and Pel concentrations were calculated by subtracting the background carbohydrate from each sample based on ePS preparations from the PAO1Δ*pel*Δ*psl* strain. Relevant levels of Psl and Pel were estimated based on prior studies on PAO1 Psl production [41].

### Neutrophil reactive oxygen species (ROS) burst assays

As previously described [42], neutrophils were infected with log phase bacterial cultures (MOI 1 neutrophil to 50 bacteria) in the presence of a luminol reporter (100μM; Sigma-Aldrich), which produces light in the presence of reactive oxygen species. Luminescence was measured every 3min for 1h with a SpecraMax M5 96-well plate reader (Molecular Devices). Phorbol myristate acetate (PMA) was used a positive control to stimulate an ROS burst response [43]. In assays using isolated ePS, 2.35μg (1x) or 23.5μg (10x) of Psl or Pel were added to each well to replicate the ePS conditions of our infections with bacterial cultures. To reduce donor-to-donor variability, ROS measurements were taken from at least three biological replicates measured in triplicate and normalized to the response generated by PMA treatment. Due to the nature of PAO1Δ*pel*Δ*psl*/pCdrAB cultures, we observed variation in inoculum CFUs despite similar OD_600_ readings. This was corrected by normalizing the AUC to the inoculum CFU. Statistical significance was determined by one-way ANOVA followed by Bonferroni’s posthoc tests. ROS time course curves were generated from a single donors response measured in triplicate and are representative of the ROS response observed among all donors tested.

### Neutrophil extracellular trap (NET) quantification

NET formation was quantified using NETosis Assay Kit (Cayman Chemical #601010) following manufacturer’s instructions. Briefly, neutrophils were infected with log phase cultures of *P*. *aeruginosa* at an MOI of 1:50 in a 24 well plate. The plate was incubated at 37°C for 4h, and free elastase was washed from the well. NETs were dissolved with DNase and NET-associated elastase was collected and quantified by comparing neutrophil elastase activity to a known standard. Three biological replicates were tested in duplicate.

### Antimicrobial tolerance

Log phase cultures of PAO1 and PAO1Δ*wspF* were pelleted and suspended in LBNS with or without 50μM H_2_O_2_, 50μg/ml LL-37, or 5% HOCl and incubated at 37°C rotating for 15min. Cells were washed once in LBNS, serially diluted, and quantified for CFUs on PIA. Three biological replicates were tested in triplicate. Statistical significance was determined by Student’s t-test.

### Cytokine quantification

Wild type (NR-9458) and TRIF/MyD88 double knockout (NR-15632) macrophage cell lines were obtained from BEI Resources, NIAID, NIH. Cells were grown to confluency in DMEM + 10% FBS at 37°C in 12-well plates. Cells were washed once in sterile PBS and infected with overnight cultures of PAO1 or PAO1Δ*wspF* (MOI 1 macrophage to 10 bacteria). After 4h, plates were centrifuged at 140x*g* for 15min and the supernatant was collected and stored at -20°C until cytokine quantification. IL-1β (R&D systems) and IL-6 (BD Biosciences) was measured via ELISA as per manufacturer’s instruction. Six biological replicates were measured in total from two independent experiments. To reduce variation between experiments cytokine levels were normalized relative to PAO1 infected macrophage levels. Statistical significance was determined by one-way ANOVA followed by Bonferroni’s posthoc tests.

### Statistical analysis

Unpaired two-tailed Student’s t-test and ANOVA with Bonferroni’s posthoc tests were employed as indicated using Prism (GraphPad v5.0 software). The threshold for significance was set at *p* < 0.05 and all experiments were repeated to ensure reproducibility of the results. Error bars in figures indicate standard error of the mean (SEM).

## Results and discussion

### RSCVs persist in a chronic wound and inhibit wound healing

RSCVs are frequently isolated from late-stage infections. We therefore used a porcine burn wound model to observe the impact of this phenotype on the host during chronic infection. Although RSCVs develop in the CF lung, current animal models for chronic pulmonary *P*. *aeruginosa* infections present a number of drawbacks. In mice, intranasal infections are typically lethal or rapidly cleared depending on the inoculum or strain utilized, but chronicity (up to 7 days) can be established by inserting bacteria embedded agar beads into the lungs or using genetically modified mouse models [44–46]. In the agar bead model, *P*. *aeruginosa* remains protected within the beads until it establishes its own protective matrix. However, the presence of agar beads causes inflammation and the extensive influx of neutrophils obstructs gas exchange often leading to complications [45,47]. The full-thickness porcine burn wound model allows us to assess *P*. *aeruginosa* chronic infections for up to 35 days [33,35].

**Fig 1:**
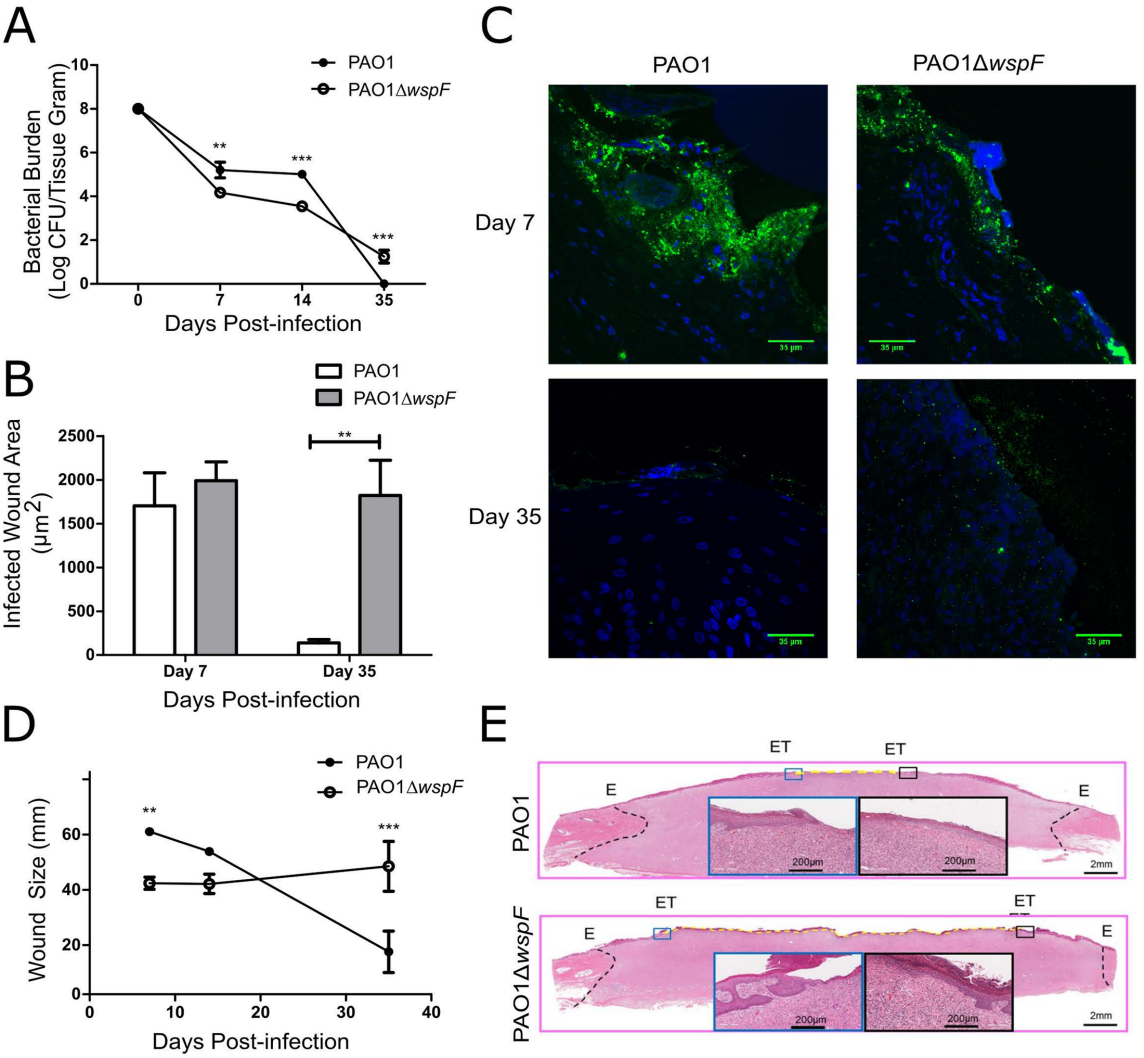
RSCVs persist during a model for chronic burn wound infection and inhibit wound healing. Porcine burn injuries were infected after 3 days with PAO1 or PAO1Δ*wspF*. A) Wound biopsies were quantified for CFUs. B and C) CLSM was used to assess bacterial burden. *P*. *aeruginosa* was stained with Alexa Fluor 488 (green) and host tissue with DAPI (blue). Area of green fluorescence was quantified. Scale bars indicate 35μm. D and E) Wound strips were H&E stained to assess re-epithelization 35 d.p.i. The distance between epithelial tongues (ET) in H&E stained tissue sections was measured. Due to H&E size constraints, images in panel D were generated by merging scans from two different slides containing half of the biopsy. Data presented as mean ± SEM. *p<0.05, **p<0.01, ***p<0.001.

To examine the impact of the RSCV phenotype during infection, pig burn wounds were infected with PAO1 or PAO1Δ*wspF*. At 7 d.p.i. and 14 d.p.i., the bacterial burden of PAO1 infected wounds was approximately 1–2 logs higher than in the PAO1Δ*wspF* infected wounds based on CFU quantification (Fig 1A). However, observation of wound cross sections 7 d.p.i. by CLSM indicated similar amounts of bacteria were present in both sets of wounds as determined by quantification of the area of *P*. *aeruginosa* fluorescent signal (Fig 1B and 1C). At 35 d.p.i. PAO1 was scarcely detectable by CFUs and fluorescence staining, while PAO1Δ*wspF* remained prevalent in the wound (Fig 1A–1C). We conclude that PAO1Δ*wspF* exhibits greater persistence in a chronic infection than PAO1, in agreement with previous RSCV studies [24–26].

Wound closure was determined by measuring the distance between the epithelial tongues (ET; leading edges of the re-epithelializing wounds) (Fig 1D and 1E). The PAO1 infected wounds showed a greater degree of re-epithelialization at 35 d.p.i. compared to the PAO1Δ*wspF* infected wounds (Fig 1D and 1E). Delayed wound healing is indicative of the severity of the infection [48], and together these observations indicate PAO1Δ*wspF* causes a more persistent and severe infection and impairs wound healing. While there are currently few reports of RSCVs isolated from wounds, these studies indicate that *P*. *aeruginosa* RSCVs can persist in the cutaneous wound environment. This suggests a broader role in persistence may exist for these variants in multiple host environments.

### RSCVs resist neutrophil phagocytosis

**Fig 2:**
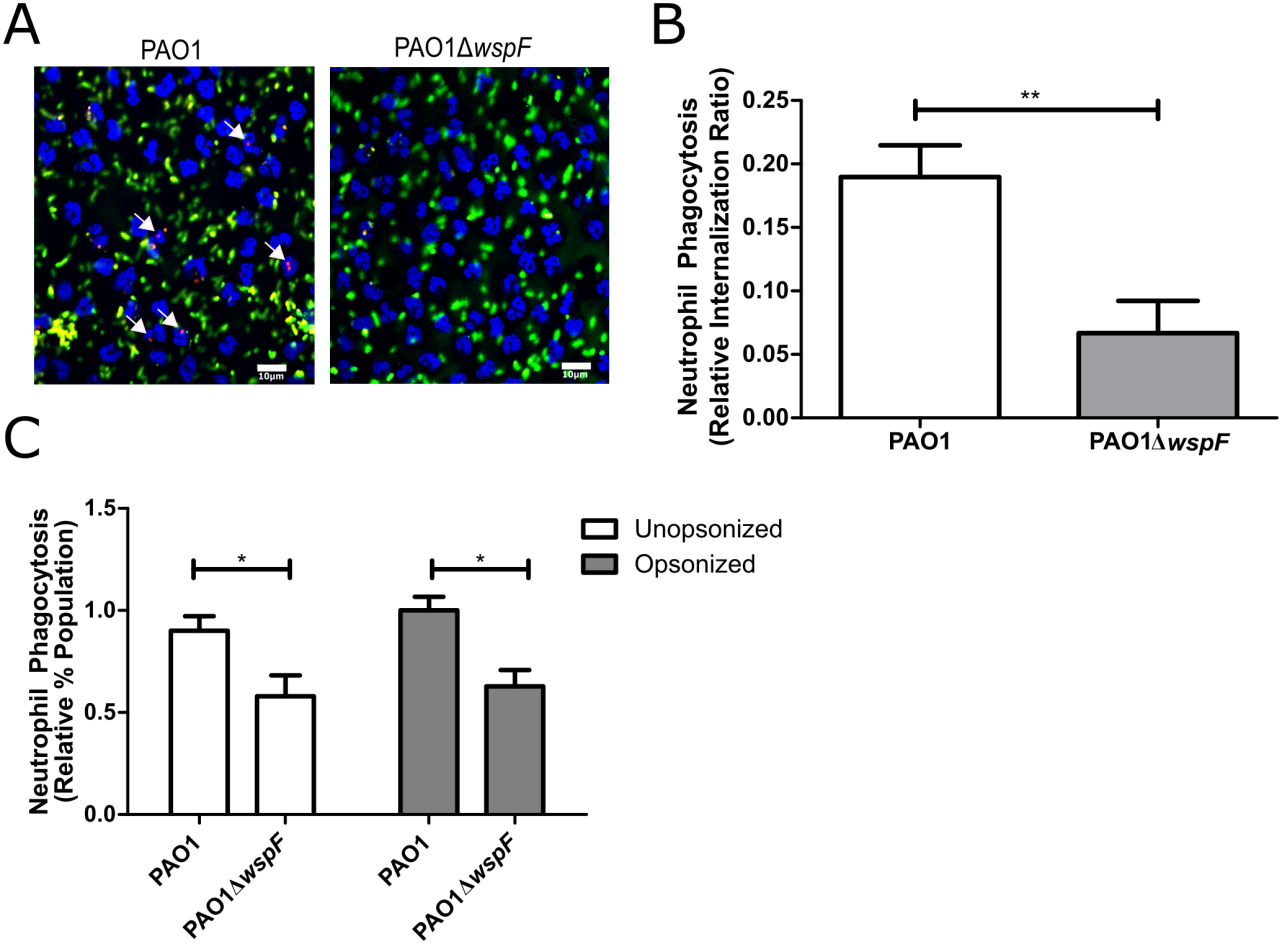
RSCVs evade neutrophil phagocytosis. A) Neutrophil phagocytosis was assessed following infection (MOI 1:50) using CLSM. Neutrophils were stained with DAPI (blue), extracellular *P*. *aeruginosa* was stained with Alexa Fluor 488 (green), and neutrophil internalized *P*. *aeruginosa* was stained with Alexa Fluor 647 (red). White arrows indicate neutrophils containing phagocytosed *P*. *aeruginosa*. B) Number of neutrophils containing phagocytosed *P. aeruginosa* was quantified and the ratio of internalization determined. C) Neutrophil phagocytosis was assessed with or without serum opsonization by quantifying the population containing GFP producing *P*. *aeruginosa* following infection (MOI 1:50). Data is presented as mean ± SEM. *p<0.05, **p<0.01.

The accumulation of neutrophils at the site of a chronic infection is characteristic of both wounds and CF lung infections [9,35,49–51]. Although abundant in these cases, neutrophils are unable to effectively clear bacteria from the host [50]. We have previously observed a predominantly neutrophil-mediated response during *P*. *aeruginosa* wound infection [35]. PAO1Δ*wspF* exhibited enhanced persistence in the wound (Fig 1), so we hypothesized the RSCV phenotype promotes evasion of neutrophil-mediated clearance. Phagocytosis is one of the primary methods utilized by neutrophils to clear a bacterial infection [52], so we began by comparing neutrophil phagocytosis of PAO1 and PAO1Δ*wspF*. Using two different methods, we observed that primary human neutrophils internalized PAO1 at a greater frequency compared to PAO1Δ*wspF* (Fig 2). The presence of bacterial opsonins, such as complement, improves pathogen recognition, phagocytosis, and bacterial clearance [52]. While serum opsonin levels in the lung environment are considered to be low [53], prior studies suggest complement factors may play an important role in the immune response to pulmonary infection [54,55]. To determine if serum opsonins influenced RSCV phagocytosis, we measured phagocytosis in the presence of human serum. Serum opsonization made no difference in the relative uptake of PAO1Δ*wspF* and phagocytosis of PAO1 remained greater (Fig 2C). These data indicate that the RSCV phenotype promotes phagocytic evasion and could provide a mechanism for persistence despite a robust neutrophil response. Furthermore, macrophage phagocytosis of the RSCV strain PAO1Δ*yfiR* was also deficient [24]. While further study will be required to understand how RSCVs avoid phagocytosis, previous studies indicate that Psl production and pathogen size can negatively impact neutrophil phagocytosis [36,56]. RSCV overproduction of ePS leads to the formation of dense aggregates, and these factors may promote phagocytic evasion and likely contribute to persistence (see below).

### RSCVs stimulate neutrophil reactive oxygen species production

**Fig 3:**
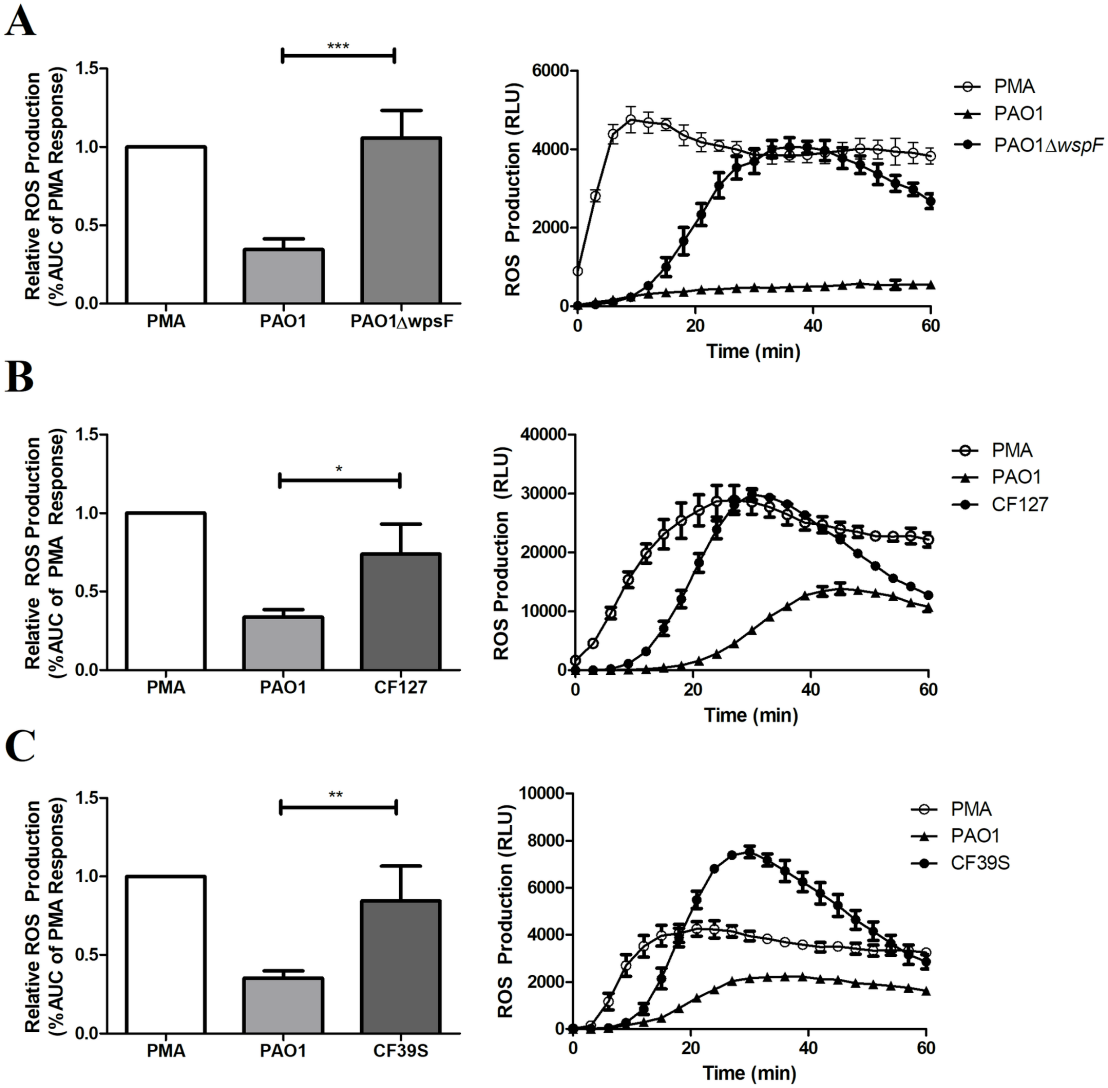
RSCVs stimulate neutrophil ROS production. A) Neutrophils were infected with log phase bacteria (MOI 1:50) or treated with PMA in the presence of a luminol reporter. Luminescence was measured for 60min (right) and the area under curve (AUC) was calculated and normalized to the PMA response (left). RLU images are representative of the ROS response from a single donors neutrophils measured in triplicate, while AUC data was collected using neutrophils from at least 3 different donors. B and C) ROS response to the CF clinical RSCV isolates CF127 and CF39s. Data is presented as mean ± SEM. *p<0.05, **p<0.01, ***p<0.001.

In response to infection, neutrophils produce a variety of antimicrobial products including ROS and antimicrobial peptides [57]. Recognition of a bacterial cell typically results in internalization, phagosome-granule fusion, and ROS promoted killing [58,59]. This process is carefully controlled, and prolonged neutrophil activity can be detrimental for the host due to damage caused to the surrounding tissue [8,50]. This phenomenon of self-induced damage has been identified in a number of chronic inflammatory diseases including CF, chronic obstructive pulmonary disease (COPD), and wound infections [8]. ROS can directly damage host tissue [60], and excessive production could explain the delayed wound healing observed during PAO1Δ*wspF* infection (Fig 1D and 1E). In this regard, we found that neutrophils infected with PAO1Δ*wspF* produced significantly more ROS compared to those infected with PAO1 (Fig 3A). ROS production was also elevated following infection with two clinical RSCV strains, CF127 and CF39s (Fig 3B and 3C). Considering the adaptability of *P*. *aeruginosa*, we expect CF39s and CF127 have acquired additional mutations during infection beyond those leading to the RSCV phenotype. While these mutations could impact the expression of various virulence factors, CF39s and CF127 exhibit common RSCV traits including ePS overproduction and autoaggregation [23,30]. Therefore, we conclude that, like PAO1Δ*wspF*, these RSCV traits elicit greater ROS production by neutrophils compared to wild-type *P*. *aeruginosa*.

Aside from direct killing, ROS production activates a variety of other neutrophil killing mechanisms, such as the formation of NETs [61]. Neutrophils can release extracellular DNA in an attempt to trap pathogens, termed NETosis [58]. NET formation also occurs following ROS production when a pathogen is too large to be phagocytosed [56]. Because we observe reduced phagocytosis but increased ROS production, we hypothesized RSCV aggregates are inducing NETosis. However, there appeared to be little difference in the quantity of NET-associated neutrophil elastase, which suggests a similar amount of NET formation occurred during infection with PAO1 or PAO1Δ*wspF* (S2 Fig).

### Bacterial aggregation promotes neutrophil activation

**Fig 4:**
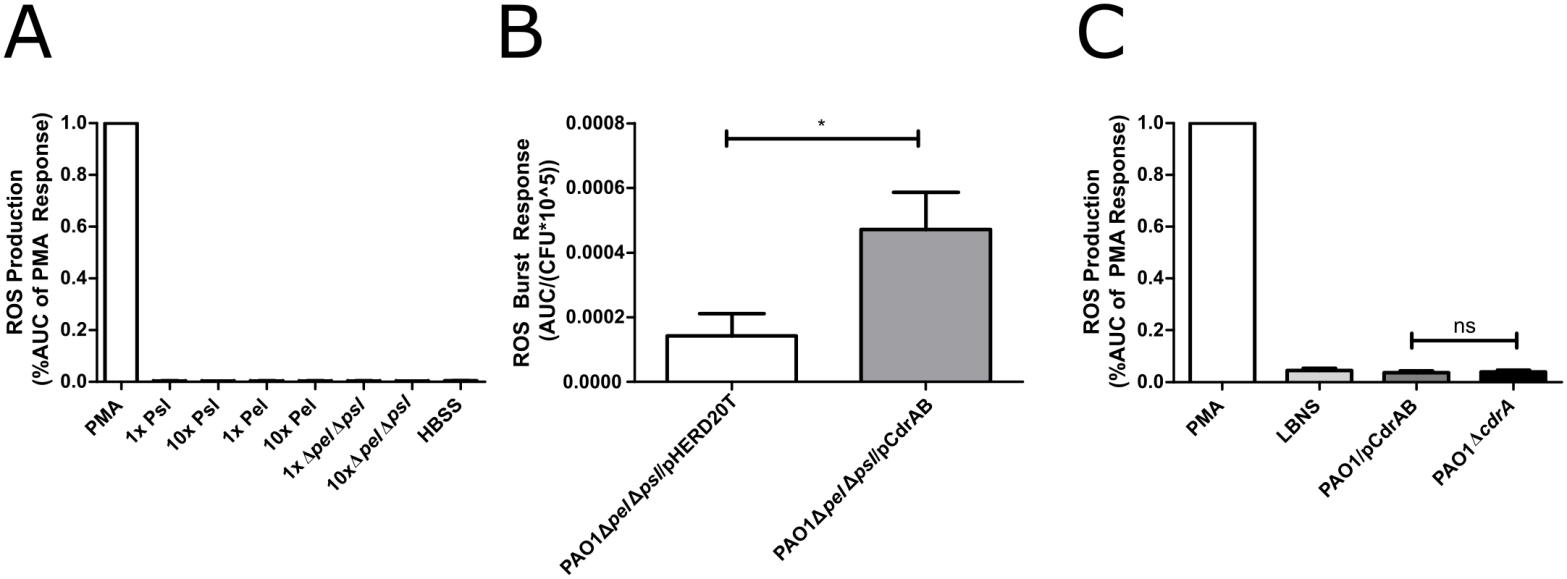
Bacterial aggregation promotes neutrophil ROS production, but exopolysaccharides alone are not sufficient. A) ePS was purified from PAO1Δ*pel*P_BAD_-psl, PAO1Δ*psl*P_BAD_-pel, or PAO1Δ*pel*Δ*psl* and total carbohydrate was quantified by phenol sulfuric acid assay. PAO1 levels or 10-times PAO1 levels of ePS was added to human neutrophils and the ROS response was measured with a luminol reporter. B) PAO1Δ*psl*Δ*pel*/pHERD20T and PAO1Δ*psl*Δ*pel*/pCdrAB were grown to log phase in the presence of 1% arabinose leading to the formation of CdrA-mediated aggregates in the strain containing pCdrAB. Primary human neutrophils were infected with bacteria at an MOI (1:50). C) Neutrophils were treated with supernatant collected from cultures of PAO1/pCdrAB or PAO1Δ*cdrA*/pHERD20T, and ROS was quantified. The AUC of the ROS response over 1h was calculated and normalized by CFUs of the inoculation culture to ensure identical cell numbers regardless of aggregation.

The RSCV phenotype stimulates neutrophil ROS production, but it remains unclear what factors specifically contribute to this response. Since RSCVs overproduce the ePS, Pel and Psl, neutrophils were treated with these components to determine if they directly stimulate ROS production. Even at high concentrations, exposure to exogenous Pel and Psl was not sufficient to stimulate ROS production (Fig 4A).

The overexpression of ePS leads to the formation of bacterial aggregates, and we predicted that, like PAO1Δ*wspF*, these structures promote neutrophil recognition resulting in more ROS production. To determine if bacterial aggregation enhances neutrophil activation, we eliminated ePS and induced aggregation by overexpressing the biofilm matrix protein CdrA [28] in the ePS mutant strain PAO1Δ*pslBCD*Δ*pelF*. The presence of aggregates was sufficient to elicit an ROS response from neutrophils despite the lack of ePS production (Fig 4B). To ensure CdrA was not directly responsible for the ROS response, supernatant containing high concentrations of CdrA was assayed, but was unable to generate an ROS response (Fig 4C). From these data, we conclude bacterial aggregation is sufficient for neutrophil activation during RSCV infection. Interestingly, a number of host factors associated with the CF lung environment, such as sputum density and neutrophil elastase activity, leads to *P*. *aeruginosa* aggregation and reduced neutrophil killing [62]. In agreement with previous reports [63,64], our studies suggest aggregation could be an important *P*. *aeruginosa* virulence factor common among all strains that contributes to persistence, and that it may be particularly important for hyper-biofilm forming strains.

### RSCVs exhibit enhanced tolerance to neutrophil antimicrobials

**Fig 5:**
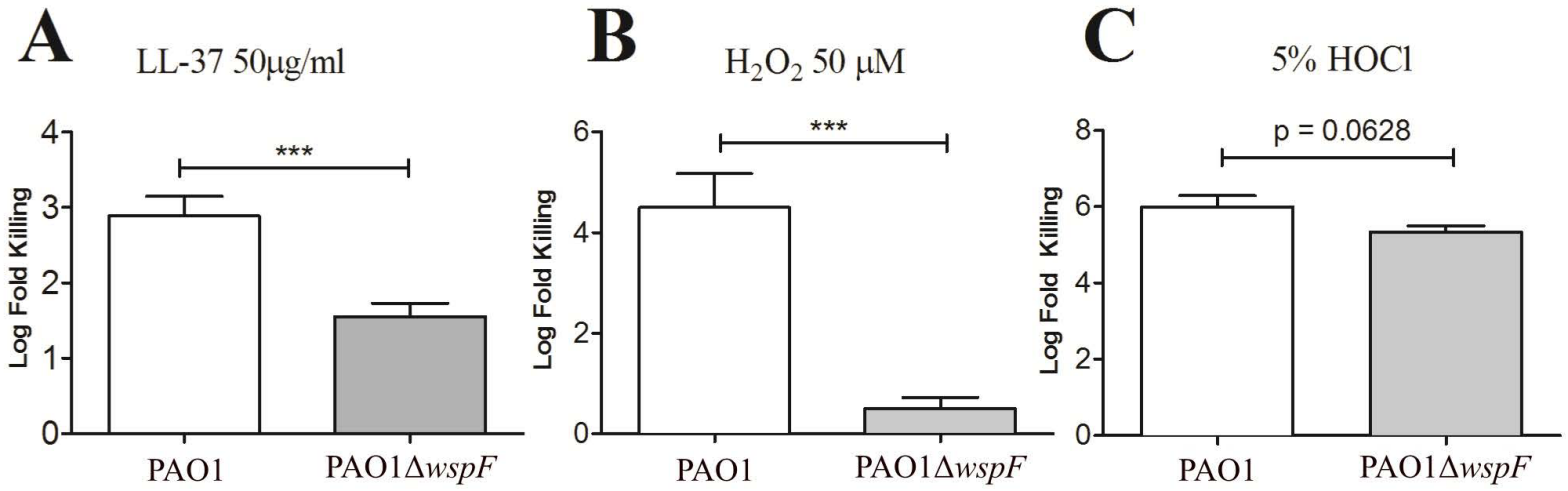
PAO1Δ*wspF* exhibits tolerance to neutrophil antimicrobial products. Log phase cultures of PAO1 or PAO1Δ*wspF* were treated with A) LL-37, B) H_2_O_2_ and C) HOCl at the labeled concentrations for 15min. CFUs were quantified before and after treatment and log fold killing determined. Data presented as mean ± SEM. ***p<0.001.

Bacterial aggregation also promotes tolerance to the neutrophil ROS compounds, HOCl and H_2_O_2_ [62]. Since RSCVs persist in the host despite a robust neutrophil and ROS response, we hypothesized PAO1Δ*wspF* would exhibit enhanced tolerance to neutrophil-produced antimicrobials. Indeed, PAO1Δ*wspF* exhibited greater tolerance to the antimicrobial peptide LL-37 compared to PAO1 (Fig 5A). PAO1Δ*wspF* was also more tolerant than PAO1 to the neutrophil ROS generating compound H_2_O_2_ (Fig 5B), and while not statistically significant, PAO1Δ*wspF* showed enhanced tolerance to HOCl (Fig 5C). A previous study determined sub-lethal doses of H_2_O_2_ promoted RSCV development primarily through loss of function mutations in *wspF* [65]. This suggests an exaggerated ROS response provoked by PAO1Δ*wspF* would not only cause host tissue damage but also further promote RSCV emergence and selection. While bacterial aggregation itself provides protection from antimicrobials [63,64], the biofilm matrix components Psl and Pel also protect *P*. *aeruginosa* [11,12,65,66]. We expect aggregation and ePS overproduction both likely contribute to RSCV tolerance to neutrophil antimicrobials and could explain how they survive despite stimulating a robust neutrophil response.

### RSCVs in the lung cause inflammation and tissue damage

**Fig 6:**
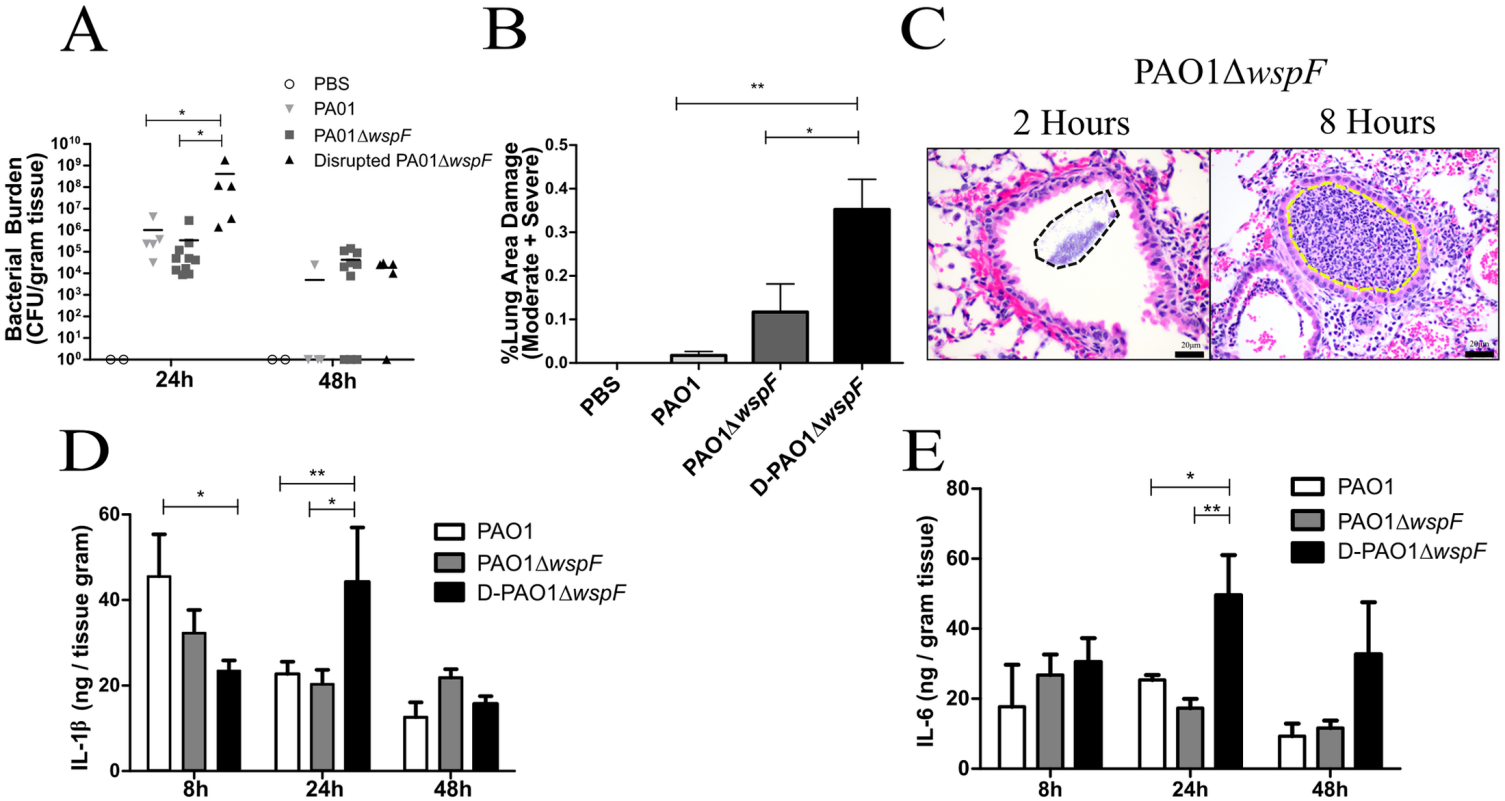
D-PAO1Δ*wspF* is hyper-inflammatory and persists during acute pulmonary infection. Mice were infected with 10^8^ bacteria via intranasal instillation. PAO1Δ*wspF* aggregates were disrupted with a 22G needle prior to inoculation as indicated (D-PAO1Δ*wspF*). A) Lungs were homogenized and CFUs quantified. B) Lungs were H&E stained 24 h.p.i. and damage was scored as minimal, moderate, or severe. C) During PAO1Δ*wspF* infection, large bacterial aggregates (black dashed area) were observed 2 h.p.i in the bronchiole spaces, and after 8h neutrophil plugs (yellow dashed area) were observed in the bronchioles. Scale bars indicates 20μm. D) IL-1β and E) IL-6 in lung homogenate was measured using ELISA. Data presented as mean ± SEM. *p<0.05, **p<0.01.

To better determine how the host responds to these variants in the lung environment, we used an acute murine pulmonary infection model. Mice were inoculated with PAO1 or PAO1Δ*wspF*, and the lungs were assessed for bacterial burden, tissue damage, and cytokine levels (Fig 6). Lung bacterial burden 2h post infection (h.p.i.) was comparable between strains ensuring similar quantities of bacteria reached the lung following inoculation (S3 Fig).

We initially observed no difference in bacterial burden or damage between PAO1 and PAO1Δ*wspF* infected lungs 24 h.p.i. (Fig 6A and 6B). However, 48 h.p.i. bacteria were only recovered from the lungs of one PAO1 infected mouse (1/5), while bacteria were recovered from the majority of PAO1Δ*wspF* infected mice (7/10) (Fig 6A). H&E staining revealed PAO1Δ*wspF* infection resulted in a highly localized colonization of the conductive zone with inflammation occurring primarily near the large bronchioles. Large clusters of bacteria were visible in the lumen of bronchiole spaces 2 h.p.i., and we observed bronchioles that were completely plugged by neutrophils at 8 h.p.i with minimal extension into the surrounding pulmonary parenchyma (Fig 6C). These neutrophil “plugs” were consistently observed during PAO1Δ*wspF* infection, and we suspect they occurred at sites of large bacterial clusters unable to disseminate to the lower airways due to size.

**Fig 7:**
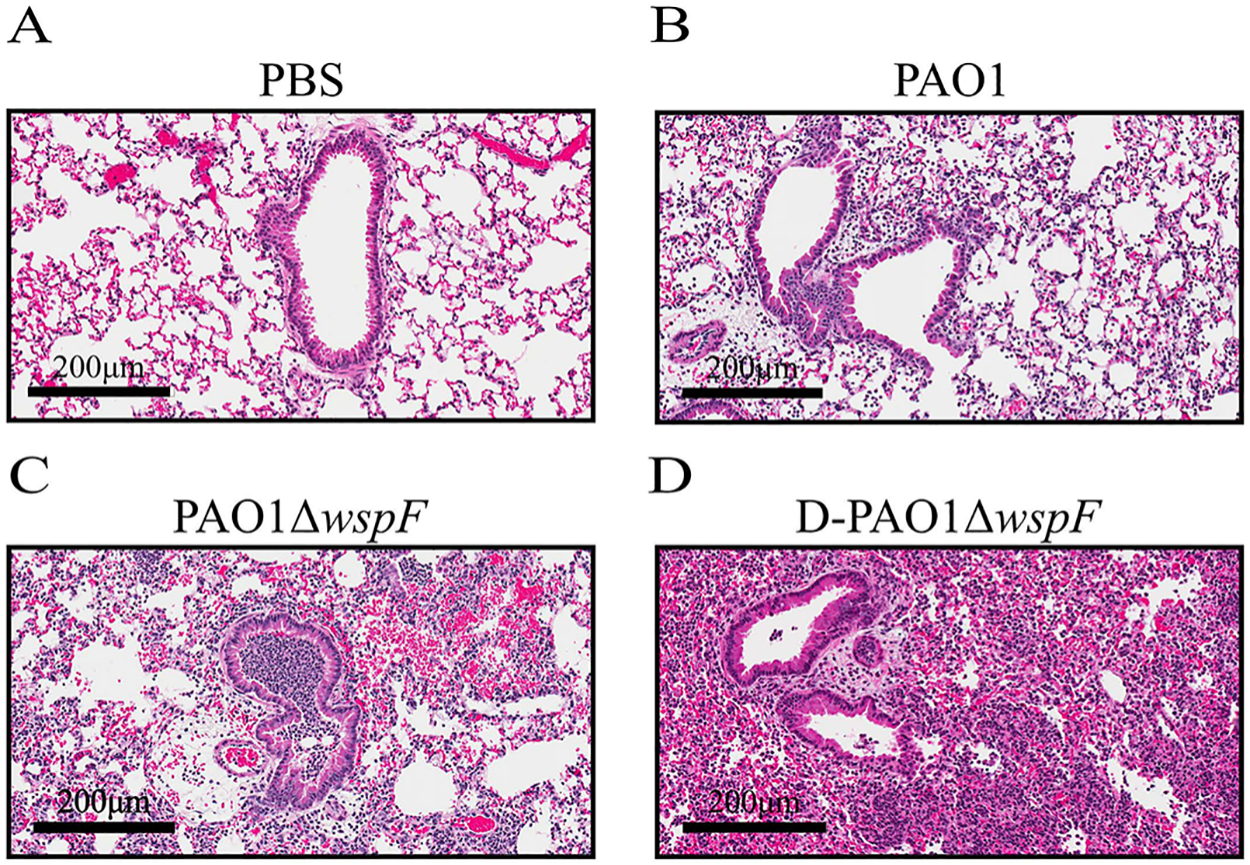
D-PAO1Δ*wspF* pulmonary infection leads to severe tissue damage and neutrophil infiltration compared to PAO1 infection. Mouse lungs 24 h.p.i. were stained with H&E to assess neutrophil infiltration and lung damage based on pulmonary architecture, necrosis, and suppurative inflammation. Images are representative of lungs treated or infected with A) PBS, B) PAO1, C) PAO1Δ*wspF*, D) D-PAO1Δ*wspF*. Scale bars indicate 200μm.

While highly variable among patients, *P*. *aeruginosa* aggregates have been observed in both the conductive and respiratory zones during chronic CF infection [51,67]. However, in our study standard intranasal inoculation methods resulted in large aggregates of PAO1Δ*wspF* primarily located within the conductive zone, while PAO1 appeared to disseminate more evenly throughout the lung. We hypothesized mechanical dispersion of PAO1Δ*wspF* aggregates prior to infection would enhance dissemination throughout the lung leading to infections that more closely resemble PAO1 inoculation. PAO1Δ*wspF* aggregates were physically disrupted prior to infection by forcing the culture through a 22G needle. Disruption of aggregates with this method was confirmed by flow cytometry (S1 Fig), and inoculation with disrupted cultures resulted in inflammation throughout the lower airways (i.e. alveolar spaces) indicating bacterial dissemination similar to PAO1 infection (Fig 7).

Contrary to infection with intact PAO1Δ*wspF* aggregates, lung bacterial burden increased during infection with disrupted PAO1Δ*wspF* aggregates (D-PAO1Δ*wspF*) (Fig 6A). This indicates the location of *P*. *aeruginosa* in the lung influences host control of the infection, where burden was lower 24 h.p.i. when bacteria were concentrated within the conductive zone. These results could explain observations from a prior lung infection study using the RSCV strain PAO1Δ*rsmA* [26]. In their study, although bacterial distribution in the lung was not directly investigated, PAO1Δ*rsmA* burden was low relative to PAO1 infection following intranasal instillation [26]. These discrepancies may be attributed in part to differences in inoculation methods, as inoculation of intact PAO1Δ*wspF* aggregates reduced bacterial burden and restricted inflammation to the conductive zone. Additionally, bacteria were detected in nearly all (4/5) of the mice infected with D-PAO1Δ*wspF* after 48h (Fig 6A), which further indicates PAO1Δ*wspF* exhibits enhanced persistence.

D-PAO1Δ*wspF* infection resulted in severe lung damage leading to loss of pulmonary architecture, necrosis, suppurative inflammation, and the accumulation of a robust neutrophil population (Figs 6B and 7). While PAO1Δ*wspF* infection of alveolar epithelial cells resulted in a subdued inflammatory cytokine response [23], we determined lung homogenate pro-inflammatory cytokine levels (IL-1β and IL-6) were elevated during D-PAO1Δ*wspF* infection compared to PAO1 infection after 24h (Fig 6D and 6E). These data indicate D-PAO1Δ*wspF* persists in the host and stimulates an inflammatory response. We hypothesize this inflammation contributes to lung damage and likely delayed wound healing (Fig 1) observed during PAO1Δ*wspF* infection. To exclude the case that the disruption process itself results in a more severe infection, we compared pulmonary infection between mice infected with PAO1 and D-PAO1 (S4 Fig). No difference in bacterial burden 24 h.p.i was observed, indicating syringe disruption does not enhance virulence.

### RSCVs activate cytokine production in a TLR-dependent manner

**Fig 8:**
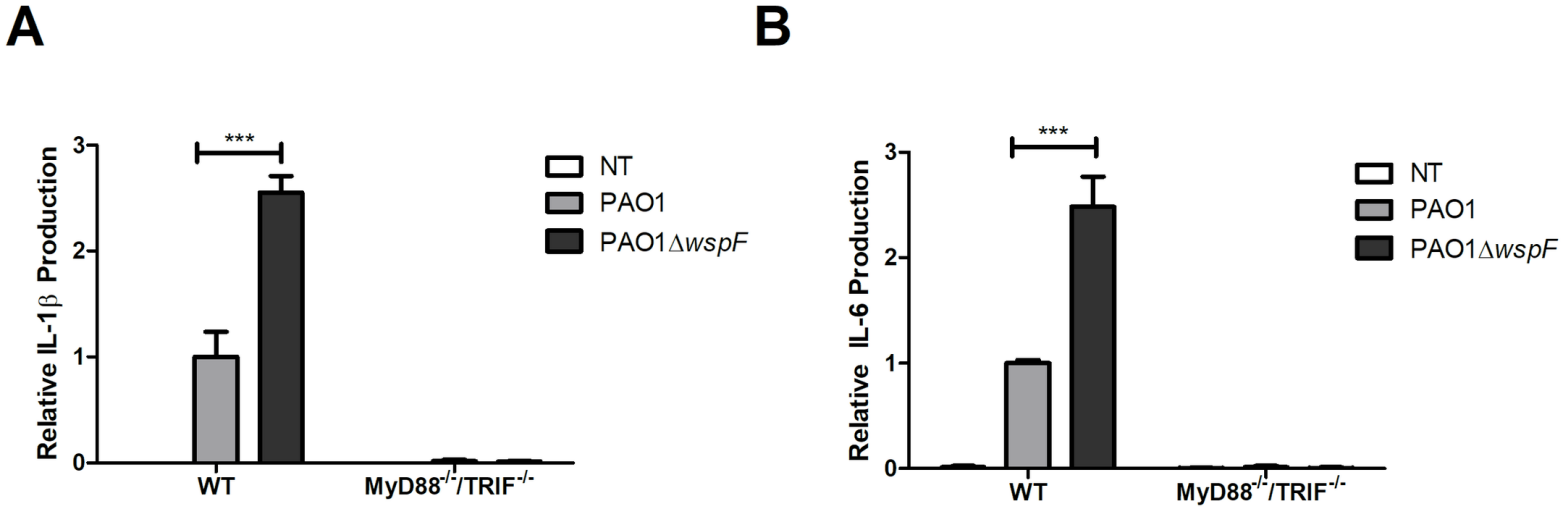
PAO1Δ*wspF* induces pro-inflammatory cytokine production in a TLR-dependent manner. NR-9456 wild type and NR-15632 MyD88^-/-^/TRIF^-/-^ mouse macrophages were infected with PAO1 or PAO1Δ*wspF* for 4h. A) IL-1β and B) IL-6 was measured in cell supernatants via ELISA. ** p< 0.01. Data presented as mean ± SEM.

Host toll-like receptors (TLRs) recognize *P*. *aeruginosa* LPS and flagellin with TLR4 and TLR5 respectively [68,69]. Macrophages, neutrophils, and airway epithelial cells initiate an inflammatory response following TLR activation, resulting in production of ROS, cytokines, and neutrophil recruiting chemokines [70,71]. In addition to LPS and flagellin, Psl-mediated attachment to host cells promotes host receptor interaction with flagella leading to cytokine production [72]. Despite producing an abundance of Psl, PAO1Δ*wspF* infection of alveolar epithelial cells resulted in a subdued inflammatory cytokine expression [23]. The authors suggest reduced expression of flagella by RSCVs results in reduced TLR5 signaling contributing to a low virulence state. However, given the extensive inflammatory response and abundance of neutrophils observed during RSCV lung infection, we evaluated the macrophage cytokine response to PAO1Δ*wspF*. Macrophage production of IL-1β and IL-6 was elevated during PAO1Δ*wspF* infection compared to infection with PAO1 (Fig 8). To determine if TLR signaling specifically was involved, MyD88/TRIF double knockouts were infected, and cytokine production was no longer observed (Fig 8). These data suggest TLR signaling has an important role in the inflammatory response to RSCV infection. We propose that greater bacterial burden due to poor phagocytosis and increased receptor interaction due to Psl overproduction may be responsible for increased TLR signaling during RSCV infection. An additional possibility is that the presence of large bacterial aggregates may increase interaction between bacteria with host TLRs.

### Conclusions

A typical CF infection begins with a single *P*. *aeruginosa* strain, which then evolves into genetically distinct subpopulations over the course of an infection [62,67]. One study classified *P*. *aeruginosa* isolates from 88 CF patients, and determined that small colony variants (SCVs) accounted for 3% of all clinical isolates which were found in 33 of the patients [22]. While SCVs readily develop in the host, it is worth noting that not all SCVs are RSCVs, and it is unlikely these variants are the only strain present during an infection. However, despite only being a small portion of the population, the presence of SCVs is correlated with poor lung function in these patients [22,73]. We propose here that, when present, RSCVs contribute to the inflammation and persistence associated with chronic infection, but it is unlikely that these strains are solely responsible for chronicity.

Phenotypic traits among SCVs can be highly variable. Reports have observed virulence factors in SCVs, such as type III secretion system (T3SS) components and siderophore production, as being up regulated or down regulated depending on the strain [26,74–76]. Yet despite this variation, up regulation of c-di-GMP, ePS production, and autoaggregation appear to be a common traits among many RSCVs [7]. Considering the *P*. *aeruginosa* genome encodes 38 proteins predicted to either produce or break down c-di-GMP [77], it is unsurprising that mutations leading to c-di-GMP dysregulation are the most commonly identified cause of the RSCV phenotype. In this study, we primarily focus on PAO1Δ*wspF*, and although our findings may only apply to RSCVs that overproduce c-di-GMP, this subset appears to be the most prevalent among SCVs. PAO1Δ*wspF* also exhibits many of the phenotypes associated with RSCVs, including increased ePS production, autoaggregation, and slow growth rate. Furthermore, comparison of PAO1Δ*wspF* to its isogenic parental strain allows us to determine specifically how c-di-GMP overproduction impacts the host response. While clinical RSCV strains could contain additional mutations that affect persistence and virulence, we anticipate our findings will broadly apply to most c-di-GMP overproducing RSCV along with other *P*. *aeruginosa* hyper-biofilm forming strains.

Collectively, our study indicates that RSCV persistence is not merely due to host evasion, and PAO1Δ*wspF* appears to utilize a variety of mechanisms to survive in the host. PAO1Δ*wspF* exhibited enhanced persistence despite eliciting a robust neutrophil response. Neutrophil-mediated inflammation is a major factor in the pathology and clinical outcome of many chronic *P*. *aeruginosa* infections, and our study indicates RSCVs may have an important role in this process. PAO1Δ*wspF* evaded neutrophil phagocytosis, activated TLR cytokine production, and stimulated an oxidative burst response, which could explain the exacerbated lung damage and delayed wound healing observed during the murine and porcine models of infection respectively. Furthermore, PAO1Δ*wspF* exhibited tolerance to neutrophil produced antimicrobials including ROS. While neutrophils derived from peripheral blood are frequently used to study bacterial infections, we acknowledge neutrophils obtained this way may not mimic neutrophils at the site of a lung or wound infection. However, the reduction in phagocytosis and inflammatory ROS response observed *in vitro* is consistent with our observations *in vivo*, and it is likely these factors do contribute to the inflammatory response to RSCVs.

RSCVs stimulate a robust but ineffective immune response that has detrimental effects for the host. We demonstrate that bacterial aggregation has a role in promoting inflammation, and likely contributes to the inflammatory response during RSCV infection. Since CF lung factors promote aggregation [64], we propose that bacterial aggregation may be an important virulence factor that contributes to the recurrent inflammation associated with the pathology of many chronic *P*. *aeruginosa* infections. Future studies focused on host immune recognition of aggregates and the role of hyper-aggregative variants will become critical for determining how to best to combat chronic, difficult to treat *P*. *aeruginosa* infections.

## Supporting information

S1 Fig RSCV culture normalization by mechanical and syringe disruption. A) CFUs of OD_600_ normalized cultures of PAO1 and PAO1Δ*wspF* following mechanical disruption by vortexing and pipetting. B) BCA protein concentration comparison between OD_600_ normalized cultures of PAO1 and PAO1Δ*wspF* following mechanical disruption by vortexing and pipetting. C) The amount of single cells in a bacterial culture was measured using flow cytometry. A low forward and side scatter gate was drawn based on the non-aggregative strain PAO1Δ*pel*Δ*psl*, which only contained single cells based on light microscopy. D-PAO1Δ*wspF* indicates the culture was forced through a 22G needle 3 times to disrupt aggregates.
(TIF)

S2 Fig PAO1Δ*wspF* does not stimulate more NETosis than PAO1. NET formation was quantified by isolating NET-associated neutrophil elastase and comparing enzyme activity to a standard curve.
(TIF)

S3 Fig Bacterial burden is similar regardless of *P*. *aeruginosa* strain at 2 h.p.i. and 8 h.p.i. during acute pulmonary infection. Mouse lung homogenate was quantified for CFUs to determine bacterial burden during infection.
(TIF)

S4 Fig Syringe disruption process does not effect infection outcome. Mice were intranasally infected with PAO1 or syringe disrupted D-PAO1 cultures. Lung bacterial burden was assessed by CFU analysis of lung homogenate after 24h.
(TIF)
