# Supplementary figures and images for: Pseudomonas aeruginosa rugose small-colony variants evade host clearance, are hyper-inflammatory, and persist in multiple host environments
Source: PLoS Pathog. 2018 Feb 2;14(2):e1006842. doi: 10.1371/journal.ppat.1006842 (PMC5812653; doi:10.1371/journal.ppat.1006842)

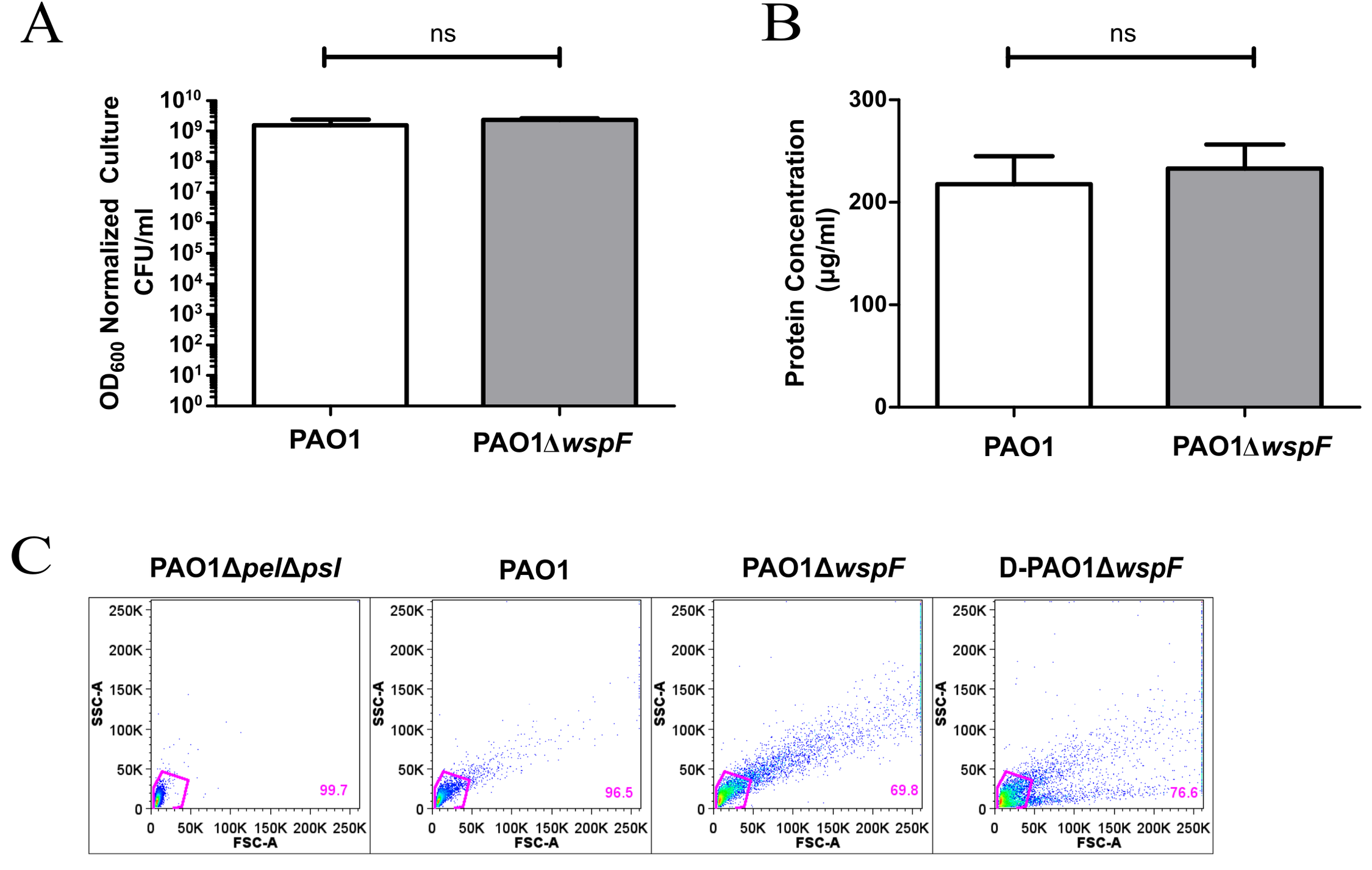

Supplement: S1 Fig — A) CFUs of OD600 normalized cultures of PAO1 and PAO1ΔwspF following mechanical disruption by vortexing and pipetting. B) BCA protein concentration comparison between OD600 normalized cultures of PAO1 and PAO1ΔwspF following mechanical disruption by vortexing and pipetting. C) The amount of single cells in a bacterial culture was measured using flow cytometry. A low forward and side scatter gate was drawn based on the non-aggregative strain PAO1ΔpelΔpsl, which only contained single cells based on light microscopy. D-PAO1ΔwspF indicates the culture was forced through a 22G needle 3 times to disrupt aggregates. (TIF) [file ppat.1006842.s001.tif]

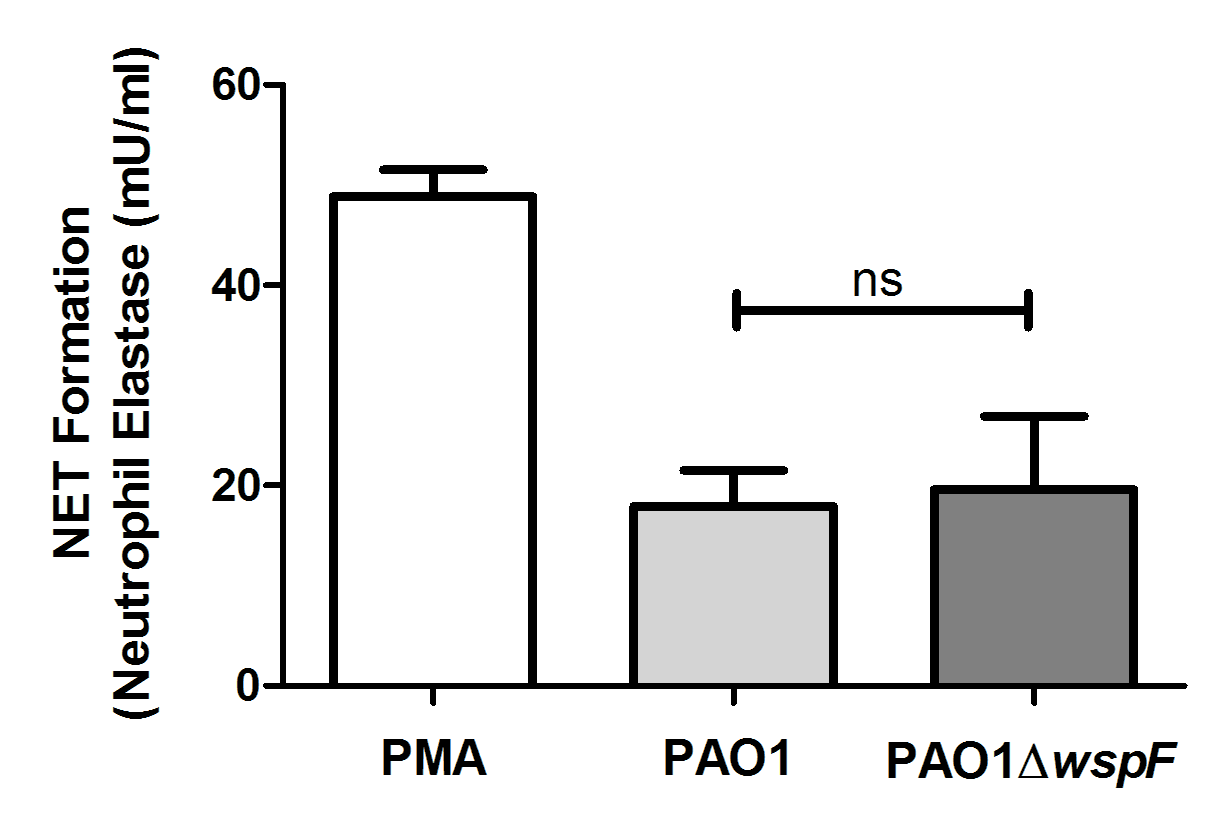

Supplement: S2 Fig — NET formation was quantified by isolating NET-associated neutrophil elastase and comparing enzyme activity to a standard curve. (TIF) [file ppat.1006842.s002.tif]

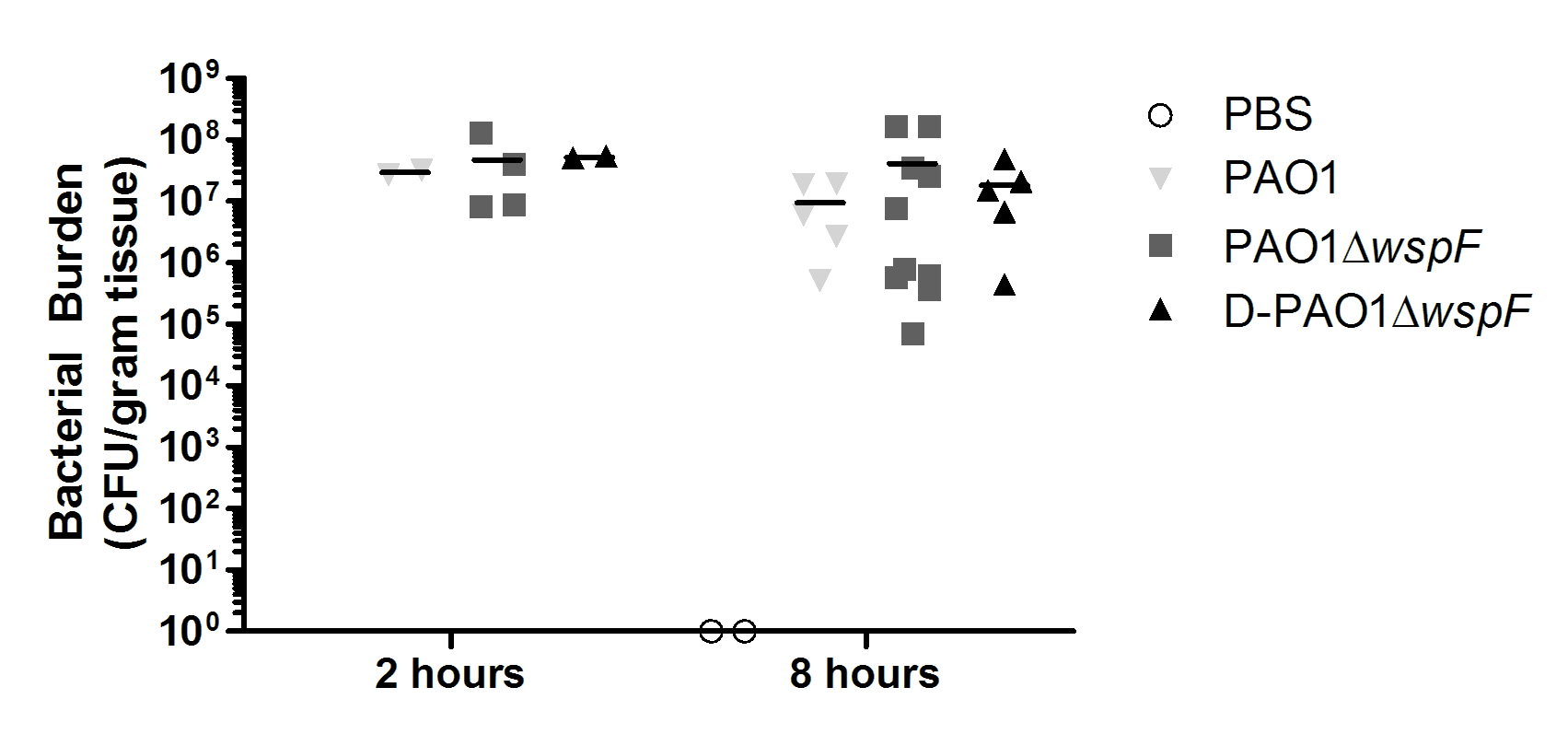

Supplement: S3 Fig — Mouse lung homogenate was quantified for CFUs to determine bacterial burden during infection. (TIF) [file ppat.1006842.s003.tif]

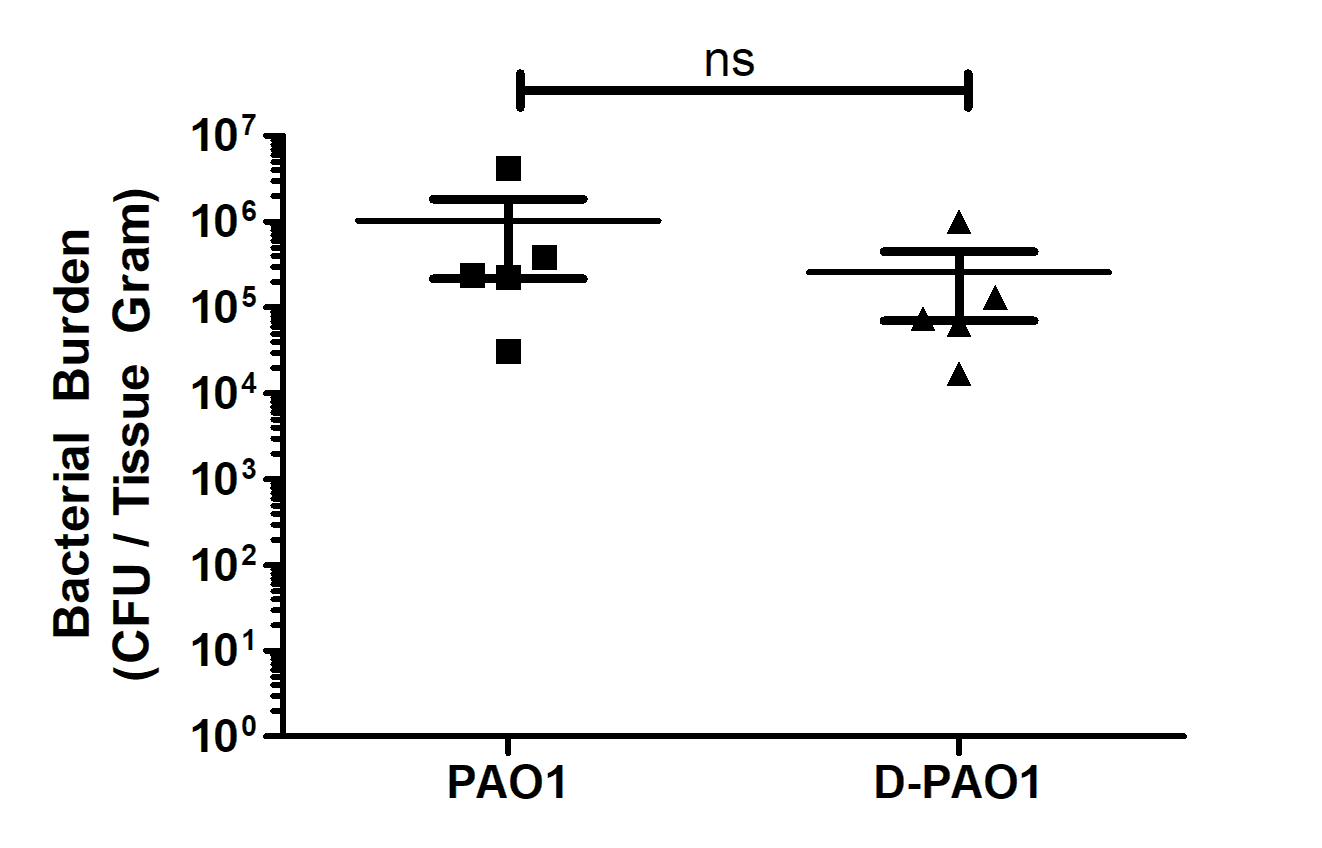

Supplement: S4 Fig — Mice were intranasally infected with PAO1 or syringe disrupted D-PAO1 cultures. Lung bacterial burden was assessed by CFU analysis of lung homogenate after 24h. (TIF) [file ppat.1006842.s004.tif]
